# Topological characterization of neuronal arbor morphology via sequence representation: II - global alignment

**DOI:** 10.1186/s12859-015-0605-1

**Published:** 2015-07-04

**Authors:** Todd A Gillette, Parsa Hosseini, Giorgio A Ascoli

**Affiliations:** Department of Molecular Neuroscience, Center for Neural Informatics, Structures, and Plasticity, Krasnow Institute for Advanced Study (MS2A1), George Mason University, Fairfax, VA USA

**Keywords:** Sequence alignment, Neuronal morphology, Multiple sequence alignment, Tree topology

## Abstract

**Background:**

The increasing abundance of neuromorphological data provides both the opportunity and the challenge to compare massive numbers of neurons from a wide diversity of sources efficiently and effectively. We implemented a modified global alignment algorithm representing axonal and dendritic bifurcations as strings of characters. Sequence alignment quantifies neuronal similarity by identifying branch-level correspondences between trees.

**Results:**

The space generated from pairwise similarities is capable of classifying neuronal arbor types as well as, or better than, traditional topological metrics. Unsupervised cluster analysis produces groups that significantly correspond with known cell classes for axons, dendrites, and pyramidal apical dendrites. Furthermore, the distinguishing consensus topology generated by multiple sequence alignment of a group of neurons reveals their shared branching blueprint. Interestingly, the axons of dendritic-targeting interneurons in the rodent cortex associates with pyramidal axons but apart from the (more topologically symmetric) axons of perisomatic-targeting interneurons.

**Conclusions:**

Global pairwise and multiple sequence alignment of neurite topologies enables detailed comparison of neurites and identification of conserved topological features in alignment-defined clusters. The methods presented also provide a framework for incorporation of additional branch-level morphological features. Moreover, comparison of multiple alignment with motif analysis shows that the two techniques provide complementary information respectively revealing global and local features.

**Electronic supplementary material:**

The online version of this article (doi:10.1186/s12859-015-0605-1) contains supplementary material, which is available to authorized users.

## Background

Neuronal morphology has been an important research topic due to its relevance to neuron growth, electrophysiology, classification, connectivity, and pathology. In recent years the availability of morphological data has dramatically increased across a broad set of species, cell types, and conditions thanks in part to search and curation efforts of NeuroMorpho.Org [1]. Meanwhile, new staining, imaging, and reconstruction technologies are producing orders of magnitude more data from animal models in projects such as FlyCircuit [2] and FlyLight [3]. Ongoing advancements are continuously improving automated reconstruction [4,5] and groundbreaking large scale endeavors such as the BRAIN Initiative are expected to expand the amount of data from mammalian organisms by additional fold factors. Importantly, these large datasets provide opportunity to validate universal morphological characteristics [6,7] as well as to discover or test features and relationships of specific neuron types [8]. Drawing inspiration from the field of genomics, where the Human Genome Project ushered in an analogous flood of data, a variety of new methods are being developed to enable search and analysis of the growing pool of morphological data.

Historically, most metrics devised for morphological analysis have focused at the whole neuron level or averaged across branches, with some derived methods quantifying a feature as a function of distance from the soma (i.e. Sholl analysis [9,10]). Pairwise comparisons have greater potential to detect fine structural distinctions, making them appropriate for search, curation, classification, and exploration of prototypical features. Recently several studies have used pairwise comparisons in morphological analysis. Pairwise sequence alignment of neurite path directional vectors helped determine the unique neuroblast lineage of larval *Drosophila* neurons [11]. The NBLAST algorithm leveraged a form of vector comparison, with nearest edges in two neuron images aligned and measured by their tangent vectors and spatial distance [12]. Thus, anatomical position and overall shape were applied for search, clustering, and classification to a database of over 16,000 *Drosophila* neurons. The Path2Path algorithm compares neurons by assigning every path, from root to tip, of one neuron to the other. The distance is given by the deformation of the paths, modulated by the difference in topological hierarchy of points along the paths [13]. An extension of the Elastic Shape Analysis Framework captures the difference between trees based on path shape and topology, as well as bifurcation locations and angles [14]. This method can also generate a representative “mean shape”, though the examples primarily represent common path features. BlastNeuron, the most recent entry into the field, focuses on aligning branches both by topology and path shape via dynamic programming after first searching for similar neurons on the basis of morphometrics [15]. In addition to providing an efficient approach for search in large databases, the alignment component could prove useful in detecting and pinpointing differences between related neurons and between reconstructions of the same neuron produced by multiple algorithms, enabling error correction and even synthesis of those algorithms.

The tree edit distance (TED) compares the topology of two trees by determining the minimum sequence of edit operations required to transform one tree into another [16]. Specifically, each branch of two trees is aligned to a branch in the other tree or labeled as an insertion. Branch features such as length, volume, surface, and bifurcation angle can be represented; in this case an edit cost based on their differences is applied for each branch assignment. The TED has been used on tree structures in multiple fields [17,18] and constitutes the most related algorithm to what we present here.

We present an original strategy to evaluate alignment of topology distinctly from other branch features across a broad range of neuronal classes. Our method exploits the novel encoding of neuron trees as sequences of characters representing bifurcations presented in the preceding companion paper [19]. We align the resulting strings with a custom-developed Python package introduced here: Pattern Analysis via Sequence-based Tree Alignment (PASTA). We used model-based cluster analysis on alignment scores to group similar neurites. Furthermore, we generated a consensus representation of clustered neurites by multiple sequence alignment revealing the conserved structural features of the corresponding trees. Sufficiently large neuron classes, well-defined by available metadata, were compared to the clusters to determine whether those classes are topologically distinct and, if so, what their defining global features are. Each arbor type of axons, dendrites, and pyramidal cell apical dendrites showed clear topology alignment clusters with distinctly conserved features. Moreover, we show that multiple alignment consensuses and motif analysis provide complementary levels of analysis of neurite topology. As an immediate application, the PASTA tool also enabled detection of previously unidentified duplicate reconstructions in the NeuroMorpho.Org database; this important curation step will be incorporated in the regular data processing pipeline of this repository. At the same time, the approach is extensible to more complex representations as required by the research goal.

## Methods

**Figure 1:**
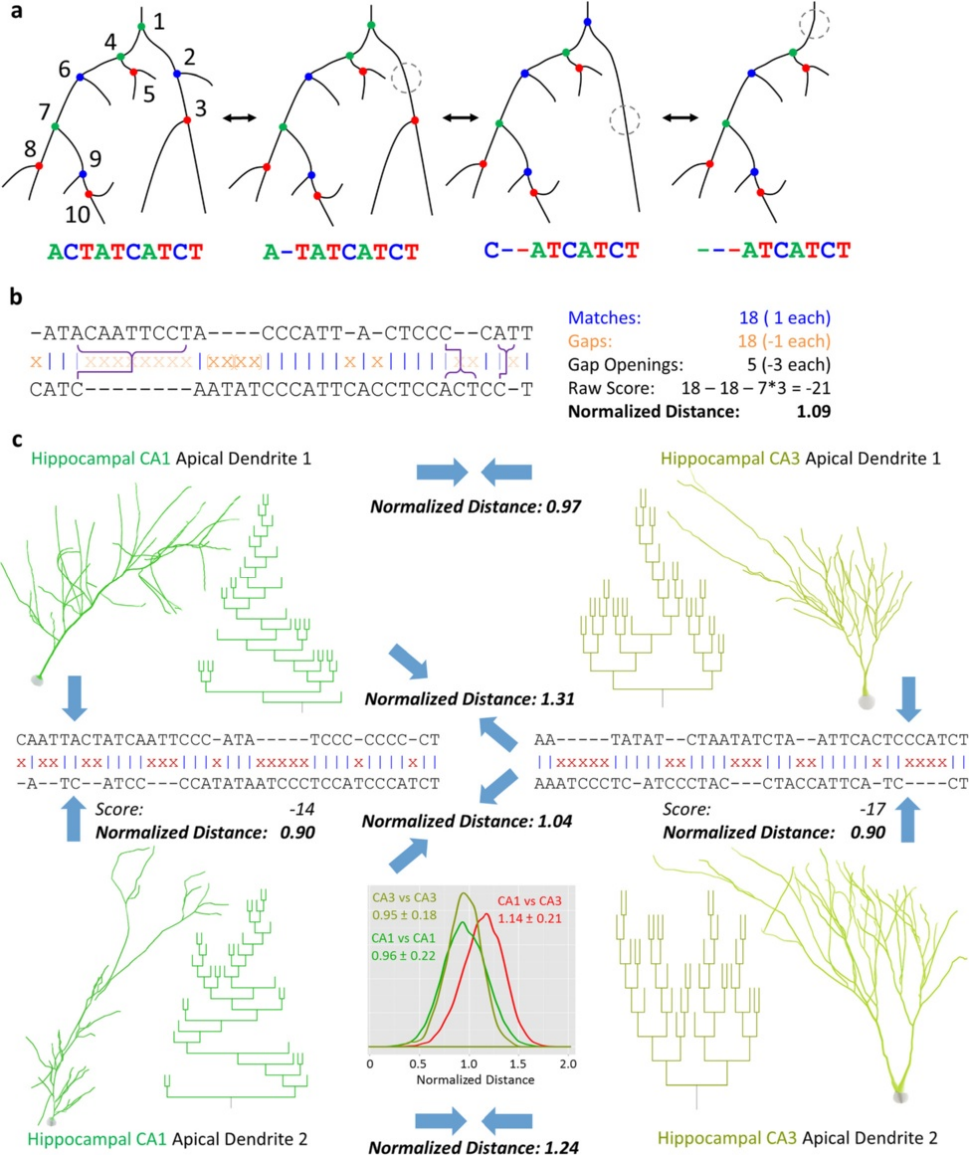
Pairwise global sequence alignment. **a.** Changes in tree structure are reflected in sequence representation. When a side-branch is lost from a non-*T* node or a *T*-node with a *C* parent, a *C* node is removed from the sequence. When a branch is lost from a *T* node with an *A* parent, the *T* is removed and the parent *A* becomes a *C*. **b.** Two tree-derived sequences are aligned by matching characters and placing gaps when an alignment cannot be made. *C* nodes or entire subtrees are gapped, from an *A* to its associated *T* (orange brackets). The *A* at the front of a gap region can be matched to a *C* (purple connected bracket). Matches (blue |) count as 1 point, gaps (orange X) count as -1 point, and openings of a gap region count as -3 points. Alignment scores are normalized and converted into distance values. **c.** Sample apical dendrite morphologies of hippocampal CA1 and CA3 pyramidal cells (NMO_00588, 07558, 00219, 00213) [51-53] and their topology dendrograms illustrate alignments between neurites of the same sub-region along with normalized distances for all 6 pairs. Raw alignment scores are also provided for within-class alignments. **Inset:** Within- and between-group distributions of normalized distances (with mean ± standard deviation) indicate that the pyramidal apical dendrites are topologically more similar to apical dendrites of their own group than to those in the other group.

Sequences are generated from neuronal trees as presented in the companion paper [19]: each branch is encoded as an *A*, *C*, or *T* depending on whether its child branches both bifurcate, bifurcate and terminate, or both terminate. A depth-first traversal keeps child branches relatively near to parent branches in the sequence representation, and traversing the smaller subtree (in terms of number of branches) before the larger (i.e. *StL* traversal) further preserves that proximity. This choice also allows for a more intuitive description of changes to a tree structure (Figure 1a).

Custom sequence alignment algorithms were developed for tree-derived sequences given their particular structural features. A global alignment algorithm was developed with specialized character types and alignment rules associated with bifurcation types. The scores produced by this method were normalized by size and a random-tree baseline prior to analysis. A multiple sequence alignment algorithm was also custom-adapted for tree-sequences from the genomics global and multiple alignment techniques.

Other well-established data manipulation and analysis methods utilized in this work are briefly described as well, such as the creation of a metric space from alignments for further analysis including classification and model-based clustering. Clusters are then statistically analyzed for associations with known cell groups. This Methods section also describes the (publicly available) dataset used in this study and related selection criteria. All code developed for this study (and associated documentation), including tree and sequence generation (Java), sequence alignment (Python), and analysis (R), is available open source at http://krasnow1.gmu.edu/cn3/NeuriteSequence/.

### Global tree-sequence alignment

Powerful bioinformatics approaches exist for comparing gene sequences to each other to determine similarity and derive evolutionary relationships. A gene may mutate by conversion of one base pair to another (edit), the introduction of a new base (insertion), or the elimination of a base (deletion). Analogous operations allow the comparison of neuronal trees: the modification of branch properties, outgrowth of new branches, and branch retraction. While neuronal trees do not explicitly share an ancestry or phylogenetic relationship typical of nucleotide sequences, they do share growth programs based on neuron type and associated genetic expression. From a functional perspective, neuronal branches have properties that impact charge transfer, signal integration, and space occupancy. The assembly plan of these features may indicate growth rules and functional attributes of the neuronal arbor as a whole.

Given the described encoding scheme [19], the growth of a single neurite branch results in one of two possible sequence edits, with branch retraction expectedly producing the opposite edit in either case. Growth prior to a bifurcation, or on the child branches of a *T* bifurcation, yields the insertion of a *C* (transition between first and second trees/sequences in Figure 1a). Growth on the terminal branch of a *C* bifurcation, however, will turn the *C* into an *A* bifurcation and insert a *T* (transition from the third to the second tree/sequence in Figure 1a). Conversely, the retraction of a *T*’s terminal branch deletes the *T* and turns the parent *A* into a *C*. If the *T*’s parent is a *C*, then the retraction effectively deletes the *C* (the *C* bifurcation becomes a *T* and the original *T* disappears). A sequence traversed by the smaller subtree first ensures that the *T* insertion/deletion occurs adjacent to the *A*/*C* conversion.

The global alignment of topological sequences is modified from the Needleman-Wunsch algorithm [20] to account for the branching structure of binary trees, with alignment rules associated with bifurcation types (Additional file 1: Figure S1). The algorithm involves a matrix created from the character positions of the two sequences being aligned running along either axis. Scores are calculated from the beginning of both sequences (top-left) to the end of both sequences (bottom-right) using previously calculated position alignments. The best score from the three possible steps to a given position is assigned to each position in the matrix (Additional file 1: Figure S1a). Those steps include a match between the characters (diagonal step from above and to the left), a gap over the character of the first sequence (a step from above), or a gap over the character of the second sequence (a step from the left). The alignment score is given in the final position and a back-trace of the optimal route from the final position to the start position produces the alignment (Additional file 1: Figure S1b).

The tree-based sequence modifications correspond to the *A*, *C*, and *T* bifurcation node types, allowing for a representation that includes branch properties in addition to local topology. It also assumes that each sequence is a single complete tree. Limitations are placed both on matching and gapping based on node type in order to ensure consistent alignment with a valid tree structure (Additional file 1: Figure S1a). Only pairs of characters of the same type are directly matched in the dynamic programming process. *A*-type characters cannot be explicitly gapped. Although this can result in inaccessible positions during back-trace, a valid back-trace will always exist as long as each sequence is a complete tree. *C*-type characters can be gapped normally. *T*-type characters can be gapped, but the gap must extend up to the associated *A* node. In terms of tree structure, the *T* node would be the last traversed node on the *A* node’s smaller subtree (assuming *StL* traversal). This within-sequence *A*-*T* relationship is defined in a recursive manner such that a *T* node’s associated *A* node is the nearest prior *A* node that does not have its own nearer subsequent *T* node. While an entire subtree can be eliminated by gapping all characters from *A* to *T*, it is also possible for all but a single branch to be eliminated, leaving a single *C* node (Figure 1b, Additional file 1: Figure S1b). Thus, when gapping a *T* node and its associated subtree, if the character in the opposing sequence position is a *C*, the gap region can end such that the *A* and *C* are matched. The final *T* node of any sequence will have no matching *A* and cannot be gapped. This would effectively lead all tree-sequences to align on the final *T* node making certain alignments impossible, such as a smaller tree aligning to an inner subtree of a larger tree. Two final modifications (included in the available code but not illustrated in the Additional file 1: Figure S1 schematic) fix this problem. First, *A* nodes can be gapped prior to any matches. Second, the final *T* node of either sequence can gap back to another *T* node that has a match.

Alignment scores are given by the number of matching positions minus the number of gaps (Figure 1b). An additional penalty of −3 is applied for each region of consecutive gaps. This cost helps distinguish more closely related sequences and reduces the otherwise high probability of a perfect score for a pair of sequences of very different lengths. Perfect scores are problematic as they produce distance values of 0, making alignment-space embedding impossible. Alternative gap opening costs were tried with no meaningful impact on the results reported here; however, tuning this parameter might help optimize future analyses depending on the specific questions of interest. Non-unitary gap costs have no impact (given the fixed scale of gap open costs) due to all matches adding the same amount to the score.

In order to focus on topological structure differences and not tree size differences, we normalized scores by sequence length. Specifically, a per-character score is computed and normalized against a length-pair matched random tree alignment baseline prior to analysis (Additional file 1: Figure S2). The normalized alignment score of a pair of trees is then converted to distance by subtracting it from 1, the maximum possible score (see Additional file 1: SM.1 for complete details).

### Spatial embedding and classification

In order to investigate the pairwise relationships among neuronal tree sequences, neurites were embedded in a multidimensional space consistent with alignment distances using Multidimensional Scaling (MDS) [21]. While edit distances would allow for standard MDS, the triangle inequality does not hold for tree alignment scores [22] or our normalized distances, thus a non-metric version was employed [23]. Instead of producing a space that preserves the original distance values in the embedded space, non-metric MDS seeks to preserve the relative proximity of the data elements. In other words, neurites that are closer to each other than to a third item in the space have that same relationship in the original data.

The embedded data were subjected to cluster analysis to determine whether different neuron classes can be distinguished in terms of their topological sequences. A wide variety of clustering methods exist, including agglomerative clustering, k-means clustering, and model-based clustering. Agglomerative clustering, including Ward’s method [24], can be run directly with distance values, obviating the need for MDS, but top level clusters are less stable than in other methods and visualization is limited. K-means assumes that the clusters are spherical and approximately of the same size [25], but neither of those assumptions is sound with regards to the diverse population of neurite shapes. Model-based clustering was chosen due to its ability to test a variety of potentially optimal model types based upon the structure of the dataset [26,27]. We used this approach to fit multivariate Gaussians with parameters for size, shape, and correlation between dimensions while varying the number of clusters. The simplest model with the greatest likelihood was selected using the Bayesian Information Criterion. Associations between clusters and known neuron classes were quantified by the relative representation of a given metadata group within a cluster and vice versa using the Bonferroni-corrected χ^2^ test (see Additional file 1: SM.4).

To determine whether the alignment space captured the same or additional information relative to that of standard topological metrics, we compared the corresponding abilities to correctly identify known neuron types using unsupervised and supervised classification. Model-based clustering is unsupervised and was restricted to two clusters for the purposes of classification. Linear discriminant analysis (LDA) is a supervised approach that produces a trained classifier based on a subset of the data [28,29]. By applying each method with different numbers of variables, an optimal classifier can be produced with the most informative variables identified. Classification accuracy was computed as the proportion of neurites in the appropriate class (additional details in Additional file 1: SM.5). Clustering performance was further measured by the adjusted Rand Index (aRI) [30,31]. An aRI score of 1 indicates perfect cluster partitions along known classification, while a score of 0 indicates random partitioning of the neurites. The metrics tested were number of bifurcations, maximum branch order, average partition asymmetry, and caulescence (main path prominence) [32].

### Multiple sequence alignment

The multiple sequence alignment (MSA) process involves several steps: the creation of a composite allowing for registration of the multiple alignment, multiple alignment of all sequences to the composite, generation of a position-specific scoring matrix (PSSM) based on the prior MSA, iteration until the MSA is stable, and finally thresholding to extract a consensus sequence (Additional file 1: Figure S3a). The composite is initially formed by aligning one sequence to another and then creating a sequence out of the alignment. Positions in which a gap exists are filled with the character on the non-gapped side of the alignment, and matches of *A* and *C* nodes take the *A* type. This process is repeated with the growing composite now being aligned to each sequence in the set one at a time (Additional file 1: Figure S3b).

The multiple alignment is formed by aligning every sequence to the complete composite in the same manner as was used in the pairwise global sequence alignment. This process identifies the sequence co-alignment positions without requiring an explicit score, thus no penalty is needed for initiating a gap region. Since the initial multiple alignment could be suboptimal, a PSSM is generated with the positions defined by the composite and the scores defined by the number of sequences aligned at each position (Additional file 1: Figure S3c). The multiple alignment is run again using the PSSM, the process iterating until stability is reached or the thresholded consensus fails to increase in size or average conservation.

Final consensuses can be determined for any given minimum conservation threshold. Higher thresholds produce shorter consensuses with higher average conservation and vice versa. A threshold of 50% was used for all MSA in this paper. In MSA columns containing both *A* and *C* nodes, the consensus value is an *A* if at least 50% of the sequences contain an *A*, and a *C* if at least 50% of the sequences contain either. The *A* node’s associated *T* will have the same count, and thus will be contained in the consensus if and only if the *A* is. This ensures that the consensus represents a single complete tree.

### NeuroMorpho.Org dataset

The dataset used in this research is the same as in the companion motif analysis [19], drawing from NeuroMorpho.Org [1] version 5.6. Briefly, it was composed of all unique neurons of the control, non-cultured condition, except for pyramidal neurons without distinct apical/basal trees. Axons, dendrites (including pyramidal basal dendrites), and pyramidal apical dendrites were treated separately, with dendrites having any terminal branches smaller than 2 microns removed to avoid confusing likely spines for dendrites. Neurites with fewer than 20 bifurcations were eliminated to ensure sufficient complexity and reduce the probability of unrelated neurites achieving high similarity by chance. A total of 6,798 neurites were analyzed, including 1,255 axons, 4,686 dendrites, and 857 pyramidal apical dendrites.

## Results

Using the separated arbors of neurons from the NeuroMorpho.Org database, each neurite was encoded as a sequence [19], aligned with every other sequence in the dataset, and the scores normalized and converted into distances (Figure 1b). Inspection of normalized distances from hippocampal CA1 and CA3 apical dendrites, two groups known to have distinct topologies, showed greater distances between sequences from different groups than from within the same group (Figure 1c). This verifies that the tree-sensitive alignment of neurite sequences can distinguish different neurite classes. While the distributions of within- and between-class distances overlap considerably, they are clearly more distinct than those generated using average partition asymmetry (Additional file 1: Figure S4), which has previously been shown to distinguish CA1 and CA3 pyramidal apical dendrites [33]. We next undertook an unsupervised cluster analysis to identify groups of neurites for inspection of topological features.

### Topology alignment space

**Figure 2:**
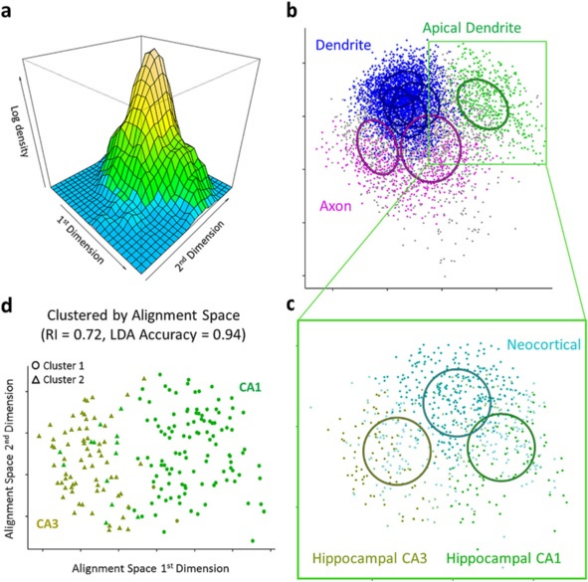
Alignment space and clustering. **a.** The log density plot of all neurite-derived sequences on the first two dimensions of alignment space suggests differentiable groups. **b.** Clustering of the alignment space reveals four clusters associated with dendrites (blue), two associated with axons (magenta), and one associated with apical dendrites (green); most neurites grouped with the cluster associated to their arbor types, but some classified with another cluster (gray). Ellipses reflect the covariance matrices of the cluster models. **c.** Sub-clustering of apical dendrites yields additional spherical clusters that associate with neocortex (cyan - top), hippocampal CA1 (green - right), and hippocampal CA3 (yellow - left). Apical dendrites improperly classified have lower color intensity. **d.** Unsupervised model-based clustering of the alignment space classifies CA1 and CA3 apical dendrites with an adjusted Rand Index of 0.72. A trained linear discriminant analysis achieves 94% accuracy.

An abstract sequence alignment space of neural trees was generated for the purposes of analyzing topological relationships between neurite types. The calculated distances were used to embed neurites into a feature space, hereon referred to as alignment space, using non-metric multidimensional scaling (MDS). Eight dimensions were sufficient for the entire dataset, with the first four showing clear structure in terms of density distribution (two-dimensional projection shown in Figure 2a). The spaces produced for each data subset are highly similar in the dominant first few dimensions which capture most of the original distance relationships. While the space is an abstraction, roughly speaking the top dimensions are mainly a function of the number of bifurcations and asymmetry. The first effect may initially seem counterintuitive since the number of bifurcations (sequence length) was explicitly controlled for; however, it is in fact consistent with the observation that dendritic branch type composition depends on tree size (see Figure four d, e in the companion paper [19]). At any rate, the correspondence between alignment dimensions and topological features is far from exact. Within a restricted region of the alignment space, moving in a set direction will result in trees with increasing or decreasing richness in some structural elements; over broader spans of the abstract space, however, the directional gradient of different elements will typically vary.

The density distribution of the alignment space suggests groupings of neurites that are more related to each other than they are to other groups of neurites. Model-based clustering methods were applied to choose the best model and the optimal number of clusters, and then to detect the clusters. Clusters take the form of multivariate Gaussians with defined size, shape, and orientation, where parameters may be the same for all clusters or unique for each.

Analyzing all neurites in the dataset yielded seven clusters with all parameters specified and distinct. Of those, four were solely associated with dendrites, one with axons, one with pyramidal apical dendrites, and one principally with axons but also significantly with apical dendrites (Figure 2b). This is consistent with the greater number and diversity of dendrites in the NeuroMorpho.Org dataset. Accuracy (proportion of neurites falling into a cluster associated with the corresponding type) was 80% for dendrites, 65% for axons, and 54% for apical dendrites due to the significant overlap with one of the axon clusters.

Each arbor type was also analyzed separately to identify potential sub-clusters and related associations with known metadata. In the case of apical dendrites, the resulting three sub-clusters clearly associated with brain regions, specifically neocortex, hippocampal CA1, and hippocampal CA3 (Figure 2c). This result highlights the importance of clustering subsets, particularly when there is a bias in proportions of available data. In some cases differences may exist but be too small relative to topological variability or have too small of a sample size to detect.

### Classification and comparison to morphometrics

In order to determine how well, relative to morphometrics, alignment space can distinguish neurites of different types, we used both model-based clustering and linear discriminant analysis (LDA) to classify neurites. In the case of CA1 and CA3 hippocampal apical dendrites (Figure 2d), the optimal set of alignment space dimensions performed (slightly) better than the optimal principal components of topological metrics (number of branches, maximum branch order, asymmetry, and caulescence) using both methods. The clustering approach produced an accuracy of 92% (aRI of 0.72) using alignment space compared to 91% (aRI of 0.67) using the principal components of the topological metrics. Alignment space performed even better using LDA, producing an accuracy of 94% compared to 89% with topological metrics.

In the case of *Drosophila* olfactory axons and mouse cortical pyramidal axons, alignment space (model-based: 87%; LDA: 87%) also produced better classification scores than topological morphometrics (model-based: 82%; LDA: 81%) using both methods. Of nine other neuronal class pairs that were at least minimally separated, the alignment space performed as well as (to within a 1% accuracy difference) or better than topological metrics in 7 out of 9 cases. These results show that, while topological metrics generally provide a robust characterization of neuronal classes, the additional detail captured by the sequence representation and alignment is sufficient to improve classification for some known classes. For the explicit purposes of classification, supplementary (non-topological) morphometrics are certainly called for; however, expanded sequence representation and pairwise alignment incorporating non-topological branch features have the potential to be more effective than those features summed or averaged across the neuron.

### Dendrite and axon clusters

**Figure 3:**
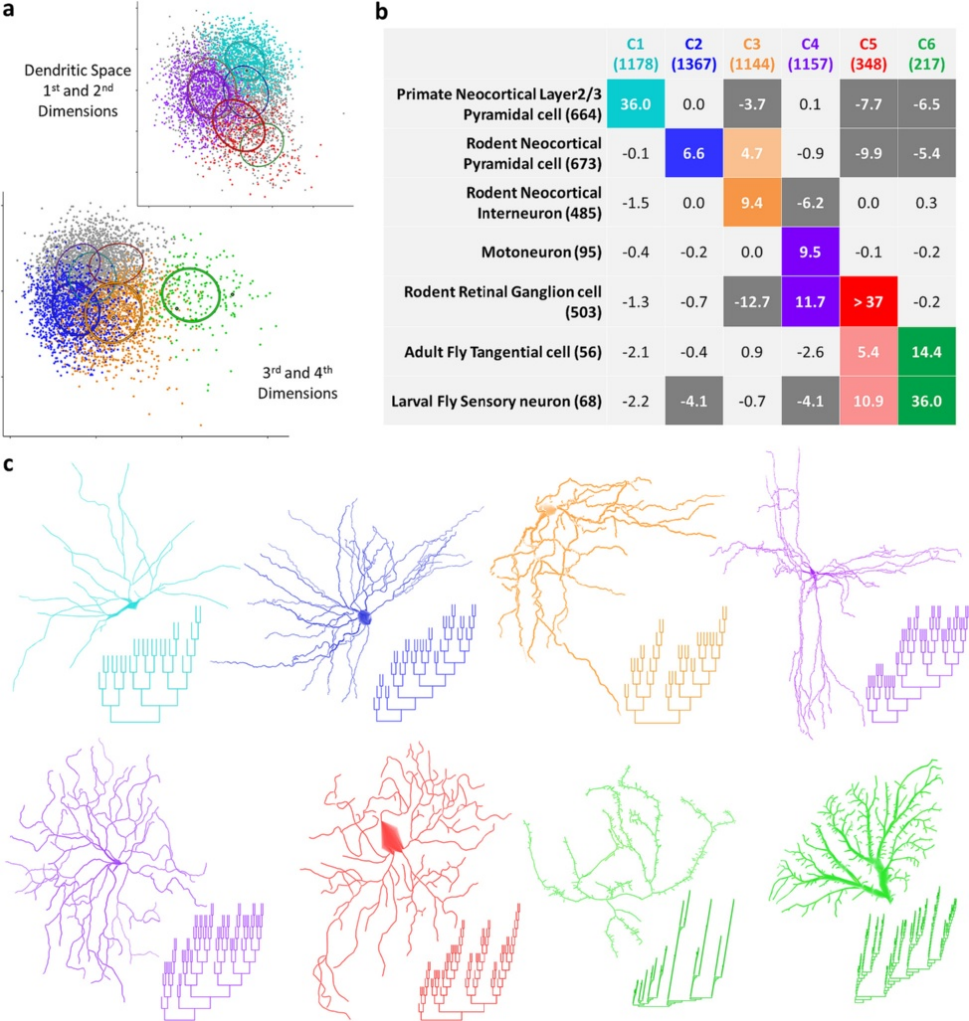
Dendrite clusters. **a.** Clusters of (non-apical) dendrites are shown in two perspectives of alignment space. Each perspective highlights dendrites in three of the six clusters, with dendrites in the other three shown in gray. **b.** The association matrix of the clusters and seven groups defined by metadata combination of species, region, cell type, and age range. The number of dendrites in each group and cluster are shown in parentheses. Values reflect the natural log of the contingency matrix p-values, with absolute values above 3 being significant (p ≤ 0.05). Dark gray cells represent cases in which significantly fewer dendrites satisfy the cluster/group association than expected given the marginals. Positive values in colored cells represent cases in which more dendrites fall into the cell than expected. Darker colors signify that an example morphology and dendrogram is provided in **c** (NMO_05022, 09439, 00298, 00625, 05409, 06531, 07043, 06659) [54-61].

We next looked at how dendrites sub-clustered by topological alignment. Dendrites, which make up the largest arbor type subset of the data (N = 5,411), fell into 6 clusters with Gaussians of varying cluster size, shape, and orientation (Figure 3a). Four alignment dimensions were required to distinguish those clusters. Upon considering dendrite classes with sufficient specificity and size, seven classes showed highly significant association with one or more clusters (Figure 3b). The 6 dendrite clusters clearly separated cortical neurons from sensory neurons of several different species. The only class that reciprocally associated with only one cluster (and with a very high level of significance) consisted of primate neocortical (layer 2/3) pyramidal basal dendrites (cluster 1). Rodent neocortical pyramidal basal dendrites alone associated with cluster 2 and jointly with rodent neocortical interneuron dendrites in cluster 3. Clusters 4–6 associated with sensory and motor neurons. Motoneurons, primarily from cat, rat, and mouse, as well as rodent retinal ganglion cells, associated with cluster 4. The rodent retinal ganglion cells associated most significantly with cluster 5 along with adult fly tangential cells (visual system) and larval fly sensory cells. The latter two groups also associated with cluster 6. Thus, the three types of sensory neurons co-clustered, suggesting that topological features are shared by neurons which respond directly to the external environment. Tree size clearly distinguishes some of the clusters exemplified in some sample dendrites from each group, but differences in the number and distribution of major branches are also evident (Figure 3c).

**Figure 4:**
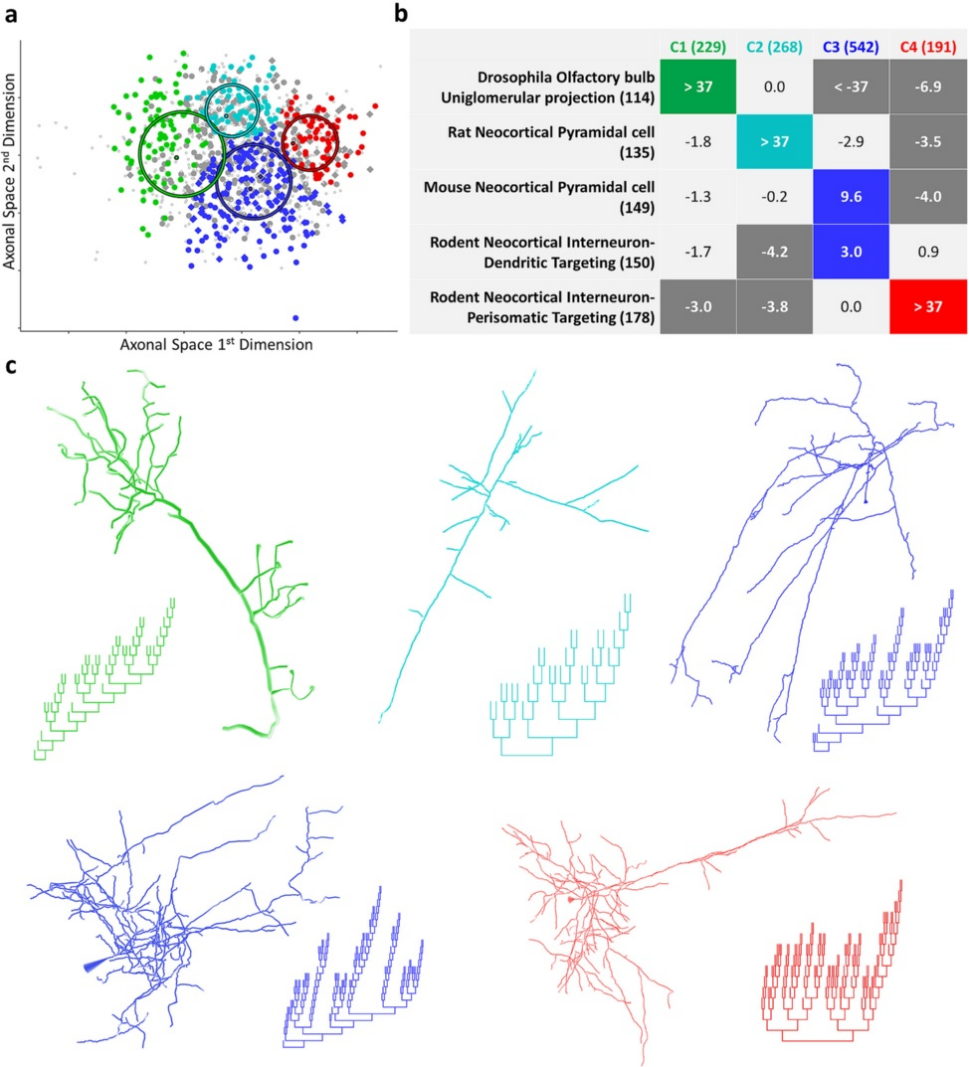
Axon clusters. **a.** Axons fall into four spherical clusters of varying sizes. Colored dots correspond to axons classified with the cluster associated with the matching metadata group. Diamonds represent rodent cortical dendritic-targeting interneurons. Circles are defined by the covariance matrix of their cluster model with a radius of one standard deviation. **b.** Association matrix for axon metadata groups and clusters. Colored and dark gray cells show significantly positive and negative associations, respectively, with the number of axons in each group and cluster in parentheses. **c.** Representative morphologies and dendrograms are shown for each significantly positive association (NMO_02574, 00315, 01209, 00424, 00424) [62-64].

Axons, though with far fewer total reconstructions (N = 1,230) and representing fewer metadata groups, tend to be larger and so have the potential to separate more clearly. Perhaps due to their relatively limited diversity, axons exhibited structure in just two dimensions and fell into 4 clusters (Figure 4a). Nevertheless, the clusters (spherical but varied in size) had strong associations with metadata groups, specifically separating insects from mammals, pyramidal cells from different rodent species, and distinct interneuron types (Figure 4b). The fly olfactory neuron axons most clearly associated with cluster 1 and are substantially dissociated from clusters 3 and 4. Rat neocortical pyramidal axons associated with cluster 2, while mouse neocortical pyramidal axons associated with cluster 3. This result should be interpreted with caution since the larger axons of rat neurons may be more prone to slicing artifacts. Since the specific histological and imaging procedures can also affect the reconstructions, it is important to note that the mouse pyramidal axons primarily come from a single archive while the rat data have substantial contributions (>10% each) from four different laboratories. Rodent (mouse and rat) neocortical dendritic targeting interneurons (primarily Martinotti and somatostatin-positive cells) also associated with cluster 3, though not as strongly. Rodent neocortical perisomatic targeting interneurons (primarily Basket and parvalbumin-positive cells) associated with cluster 4. Representative morphologies and dendrograms (Figure 4c) suggest features which are shared within or even define each cluster. Derivation of consensus representations and the qualitative and quantitative description of those features follow.

### Multiple sequence alignment and consensuses

**Figure 5:**
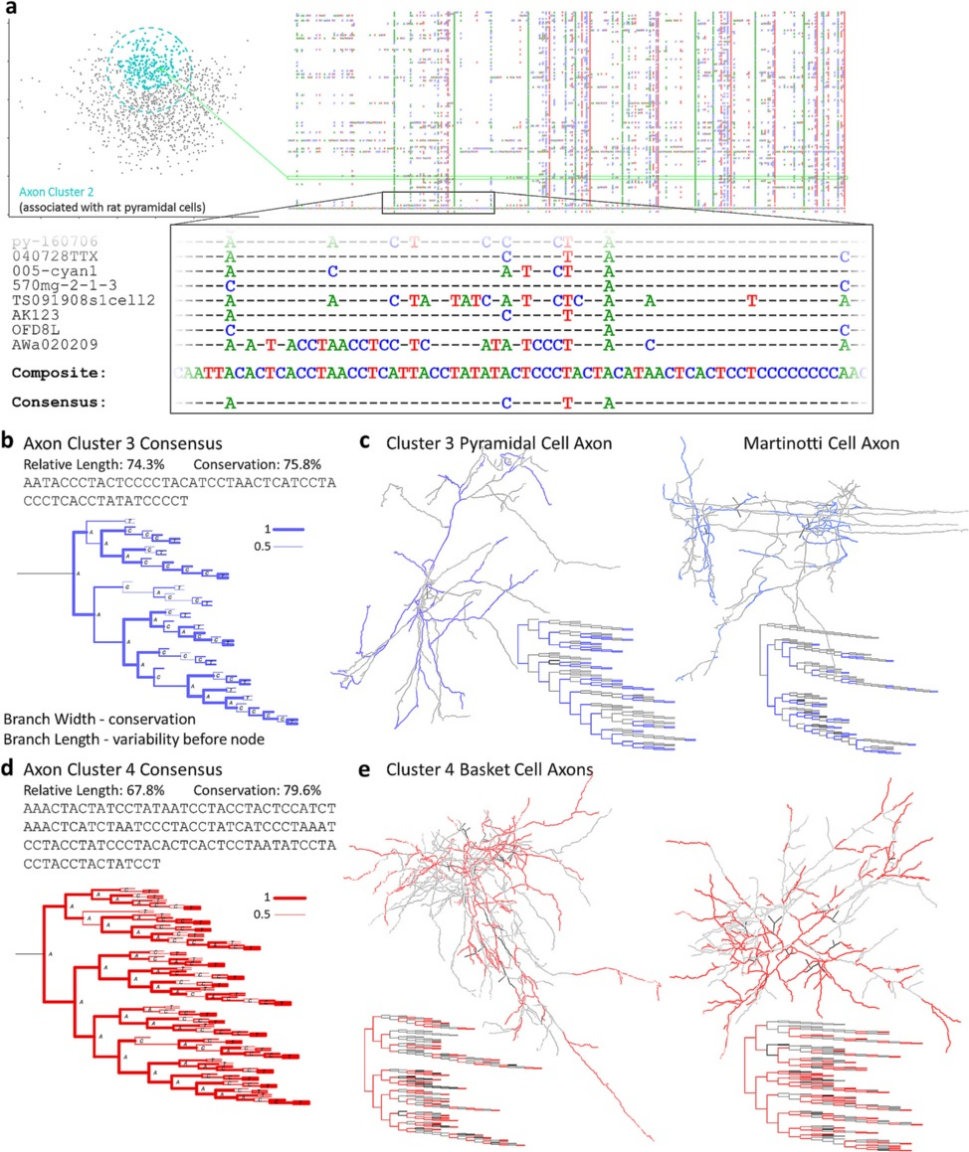
Multiple sequence alignment and consensuses. **a.** Sequences of axonal cluster 2 (left) are aligned (right). A section of the alignment is magnified along with the composite and consensus (bottom). A single neurite in the cluster and its sequence in the MSA is highlighted (green). **b, d.** Consensuses of axon clusters 3 (**b**, blue) and 4(**d**, red) in sequence and dendrogram form, with relative length and average conservation statistics. Dendrogram branch width indicates conservation of the parent bifurcation (from 0.5 to 1). Branch length indicates variability preceding a bifurcation, calculated as the average proportion of a sequence falling between conserved bifurcations. **c, e.** Example morphologies and dendrograms of cluster 3 (**c**) and 4 (**e**) (NMO_02624, 00427, 07447, 00332 from [41,63,65]). Colored segments indicate an alignment with the consensus at the bifurcation. Black and gray segments indicate consensus bifurcations not found in the neurite and vice versa.

Multiple sequence alignment (MSA) provides the means for extracting common structural features of a group of neurite sequences and measuring their level of conservation. MSA can be visualized as vertical densities illustrating how represented various positions are (Figure 5a). A consensus structure can be defined by setting a threshold for the proportion of member sequences aligned at each position. A low threshold will result in a relatively larger consensus with lower average conservation (matching bifurcations) across member sequences. This choice tends to reveal common local patterns, though in the extreme, motif analysis [19] could be more effective for that purpose. Higher thresholds, in contrast, reveal the common global pattern by producing narrower consensuses with higher average conservation. A 50% threshold was found to yield consistently clear results across clusters. In general, the relative consensus length (number of bifurcations normalized by the median sequence length in the group of neurites) and average conservation together provide a measure of how inter-related the sequences in a set are in terms of shared topological features. For a measure of global representativeness that accounts for the sequence length variability, we also employed the normalized pairwise alignment score.

We take for comparison axon clusters 3 and 4, which are next to each other in alignment space but display considerably different consensus features. In order to focus on the most representative trees of each cluster, we only considered those sequences within one standard deviation of their cluster centers. Furthermore, to avoid any potential effect of number of sequences on the MSA, the 120 sequences (the smallest extracted set size, from cluster 4) closest to their respective cluster centers were used for MSA and subsequent analysis. The consensus of cluster 3 has an absolute length of 52, a relative length of 74.3%, and an average conservation of 75.8% (Figure 5b). The consensus of cluster 4 has a substantially longer absolute length of 117 but a smaller relative length of 67.8% due to the larger median sequence length relative to cluster 3 (Figure 5d). The cluster 4 consensus also has a higher average conservation of 79.6%, meaning that while cluster 3 has a higher proportion of well-aligned branches, the well-aligned branches of cluster 4 are more densely represented in the cluster sequences.

These properties are in part explained by the different variability of sequence length between the two clusters. If the higher variability of a cluster is due in part to a larger number of relatively short sequences, fewer bifurcations overall could align in the consensus. Longer sequences may be more likely to contribute to the consensus but will also align in many positions that do not pass the consensus threshold. Cluster 3 sequences have an average length of 139.7, median of 70.5, and standard deviation of 173.3; in contrast, cluster 4 sequences have an average length of 193, a median of 174, and a standard deviation of 98.8. The impact of this variability is seen in the branch lengths of the consensus dendrogram (Figure 5b, d). However, alignment scores normalized to z-scores (a score of 0 being equivalent to an average alignment between random sequences), show the same relationship with cluster 3 having a score of 1.42 ± 0.06 compared to the cluster 4 average of 2.77 ± 0.09 (p = 6.7e-8). This is consistent with the larger Gaussian model which was fit to cluster 3, indicating the looser relationship between its sequences. The consensus dendrogram of cluster 3 shows where a substantial portion of the variability falls. The root branch is particularly long, meaning that a relatively large proportion of parts of member sequences exist prior to that bifurcation. Sample axons from the cluster illustrate the alignment of the axons and consensus in full context (Figure 5c).

Consensus structural details also differ substantially between clusters 3 and 4. Consensus 4 has greater symmetry (Figure 5d): in fact, consensus 3 has a maximum branch order of 13 compared to 12 for cluster 4, even though cluster 4 has 65 more bifurcations. The axons of cluster 3 show a greater average asymmetry of 0.611 compared to cluster 4 axons asymmetry of 0.54 (p < 2.2e-16). Other structural differences could relate to differential spatial targeting of these classes. Although the arbors from the two clusters showed similar spatial arrangements (measured by the distribution of weighted Euclidian distances of branches from the arbor center of mass), their total neurite length differed significantly. Cluster 3 had a median length of 10,050 μm (mean 14,530 μm) and cluster 4 a median of 12,952 μm (mean 19,450 μm; Wilcoxon rank sum test; p < e-13). Although many of the larger neurons in the clusters were not from the associated cell classes, the trend was consistent. Pyramidal axons, with a median length of 3,261 μm, were shorter than dendritic targeting interneurons, with a median length of 5,440 μm (p < 1.6e-15). Perisomatic targeting interneurons, with a median of 6,671 μm were yet longer (p < 2.0e-3). The greater length in spite of similar spatial arrangement suggests a greater density or some alternative heterogeneous distribution of branches for cluster 4 axons.

### Consensuses and motifs

**Figure 6:**
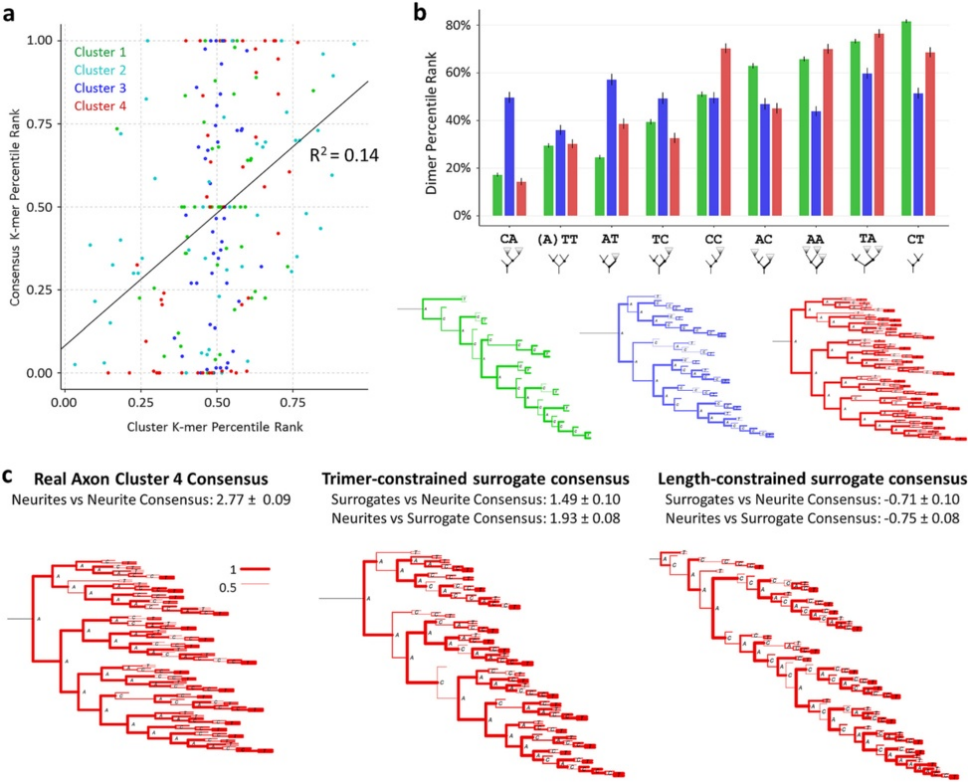
Global consensus and local motifs. **a.** Plot of the *k*-mer percentile ranks (normalized frequencies), for each dimer and trimer of every cluster, with the cluster’s consensus values on the y-axis and the cluster’s average sequence values on the x-axis. The small correlation indicates that consensuses only capture a small proportion (14% of the variance) of the local motif information about the cluster’s members. **b.** Dimer profile (top) and consensus dendrograms (bottom) for clusters 1, 3, and 4. The dimer profile is similar between clusters 1 and 4, while the consensuses differ dramatically. **c.** Consensus dendrograms of cluster 4 neurite sequences, trimer-constrained surrogates of the sequences, and length-constrained surrogates of the sequences. Average normalized alignment scores are provided given a set of sequences aligned to a single consensus. Dendrogram branch widths show parent bifurcation’s conservation.

We next considered whether the consensus is representative of local motifs patterns [19], and whether motifs capture the global features of the consensus. Trimer-constrained surrogate sets were generated for each axon cluster consensus and for each individual axon sequence in order to produce motif profiles for comparison with the motif profiles of each cluster’s sequences. Briefly, random trees were generated with just size, size and node-type proportions, and size and dimer proportions equivalent to each consensus. For a given consensus or axon sequence, node-type, dimer, and trimer proportions were then normalized to the respective proportions in the surrogate sets by determining the percentile rank of a given consensus *k*-mer proportion within the surrogate set’s *k*-mer proportion distribution (see companion paper [19] for further details). The correlation between the motif patterns in the neurite sequences and their corresponding consensuses was small across the 4 axonal clusters (Pearson coefficient of 0.37), with the consensus values varying substantially more than the cluster averages (Figure 6a). Moreover, while clusters 1 and 4 had very similar motif profiles, with only minor differences in dimers *AT*, *CC*, *AC*, and *CT*, their consensuses differed dramatically (Figure 6b). For further comparison, cluster 3’s values are very different from clusters 1 and 4, falling much closer to baseline, while the global consensus appears intermediate between those of clusters 1 and 4. Thus, while differences in global structure can in principle influence motif patterns, local structural features appear to be largely independent.

Motifs were similarly insufficient to consistently capture global features. Trimer-constrained surrogates of each cluster were aligned and consensuses generated. Some trimer-constrained surrogate consensuses were comparable to their real neurite consensus counterparts. For instance, global alignment scores of cluster 3 neurite sequences aligned to the neurite consensus were not significantly different than the scores when aligned to the surrogate consensus. However, in other cases, most notably cluster 4, the trimer-constrained surrogates produced a far less representative consensus (Figure 6c). For comparison, length-constrained surrogates always failed to produce representative consensuses with alignment scores systematically below those achieved with trimer-constrained surrogate consensuses. This means that generally motifs and consensuses have only limited impact on each other and they provide complementary information relating to local and global patterns, respectively.

## Discussion

The following discussion focuses specifically on sequence alignment. More generally relevant issues of topological encoding are discussed in the companion article [19].

### Biological interpretations and current limits of experimental data

Axons are of particular interest due to their role in connecting neurons to enable network function. Given the diversity of cortical inhibitory interneurons in terms of axonal morphology [34], it is not surprising that these cells would fall into different topological clusters. Perisomatic-targeting interneurons are responsible for generating synchronous network rhythms [35,36] by densely synapsing on a large number of principal neurons. The relative symmetry of perisomatic-targeting interneurons might then reflect a combination of dense synaptic fields along with growth mechanisms to ensure minimal path distance (balanced against wiring minimization) [37]. Shorter paths to targets would serve to optimize synchrony of the GABAergic signals from the interneuron.

It is interesting that a complementary subset of (dendritic-targeting) interneurons would instead associate with neocortical pyramidal cells based on the topological sequences of the respective axonal arbors. This does not mean that the two axonal classes have the same distribution of topological features, as they do not share the cluster space equally; however their co-clustering does suggest a substantial overlap in those features. The relatively asymmetric branching of dendritic-targeting interneurons and mouse pyramidal cells suggests two possible non-exclusive explanations. The first is that the two classes are similar in their spatial targeting, and distinct from that of perisomatic-targeting interneurons. An alternative is that both classes are not as highly constrained in the timing of their efferent signals and thus wiring minimization is preferred over path minimization and its limiting effect on signal delay. The spatial targeting explanation is consistent with the roles of pyramidal cells and dendritic targeting interneurons in network computation requiring a diverse synaptic distribution to generate more powerful networks [38,39]. Specifically, sparser postsynaptic targeting would yield greater differences between the connectivity profiles of principal neurons and thus increase network complexity, which may aid in computational functions such as pattern separation [40].

The differentiation between mouse and rat pyramidal cell axons is not as clear from a biological perspective. It is important to recognize, however, that most morphological data are derived from in vitro slice preparations, most often preventing the full reconstruct of axonal trees. Relative to dendrites, axons typically involve more expansive arbors and span well beyond the region explored in a given experiment. Given the size difference between mouse and rat brains, the impact of slicing on the reconstruction would be larger for rat axons. To test this hypothesis, we compared artificially clipped mouse pyramidal axons to their rat counterparts. Our analysis suggests that morphological incompleteness due to sectioning artifacts is not the source of the difference between mouse and rat. Three pyramidal cell axons of various sizes (155, 495, and 1,725 bifurcations), were selected among the few instances of in vivo (putatively more complete) reconstructions, one from rat hippocampus and two from cat neocortex. To simulate the effect of tissue slicing, these arbors were modified by eliminating all branches and sub-trees beyond a given distance in the z-dimension from the soma. Cutting at several distances showed a decrease in normalized alignment score, but even with a quarter of the z-range and less than 50% of the original sequence length, the scores always remained significantly greater (four standard deviations above the mean) than length-matched random sequences. Other similarly severe cuts maintained much higher normalized scores. This suggests that sequence analysis is relatively robust to reconstruction artifacts of this kind. Thus, brain size is not likely to explain the differences between rat and mouse axonal trees. Other experimental protocol details, or an unknown biased sampling of neocortical pyramidal neurons, may yet be responsible; however, it is also possible that mouse and rat pyramidal cell axons indeed slightly differ in their branching topology.

### Sequence alignment and expanded representations

The global sequence alignment modifications were necessary to ensure proper accounting of tree topology. Attempts to use standard global alignment (not respecting tree structure) and a simpler clustering method revealed no meaningful differences among known neuron types. This is because very different trees could have similar sequences if the structural attributes of bifurcations are disregarded. For instance, three subsequences with distinct patterns, α, β, and γ, could be arranged in sequence as αβγ and matched to another sequence α’β’γ’ where α’ is a close match to α, β’ to β, and γ’ to γ. These two trees, however, could be arranged in very different ways: α as the parent of subtrees β and γ, and α’ as the parent of β’ and β’ as the parent of γ’. Thus, αβγ and α’β’γ’ would match well despite substantially different tree structures. In contrast, the modified global alignment algorithm implemented in PASTA distinguishes the two cases.

Expanded representations including branch-level morphological features could enhance alignment sensitivity, decreasing the likelihood of spurious alignment for shorter sequences. For instance, several characters could be used for each topological node type to encode the bifurcation angles (i.e. between child branches, between bisector and parent branch, or between child plane and parent) relative to the population distribution. The increased sequence complexity would alleviate the need for a gap opening cost, allowing a greater range for parameter tuning. Additionally, such encodings would enable analyses for relating local topological patterns with other morphological features. Subtree size and tortuosity are similarly reliable branch metrics which could be encoded; distance from soma and maximum distance to termination are also suitable candidates. Diameter and associated metrics (e.g. surface area and volume), while possible in principle, are more susceptible to experimental protocols [33]. Combining several morphological attributes in the encoding would multiply the number of characters required, but feature vectors could be used rather than characters, increasing sensitivity at the expense of speed. Alternatively, the number of features and their discretization could be judiciously selected based on the specific research questions.

Leveraging pairwise tree alignment, cluster analysis, and multiple sequence alignment, an expanded encoding could help discover novel relationships between morphological features, including their relative conservation and specificity across neuronal classes. For instance, the different tortuosity among interneuron classes [41] could reveal regional differences within the arbor, which would suggest axonal domains with specific targeting features. The level of conservation would provide an indication of the feature’s functional importance as well as provide a constraint for modeling growth mechanisms.

Since morphology ultimately depends on ultrastructural features to produce function, lower-scale branch properties such as spines or bouton densities and even local molecular expression could create powerful analyses. This would require a richer data type and new experimental processes, but with recent technologies the prospect is real [42-44]. Combining biophysical data with pairwise alignment would enable exploration and detection of morphological-physiological relationships, such as spike failures near regions of high bifurcation density, or receptors of neurotrophic factors in sequences with many *C* nodes. Spatial information, such as the cortical layer or brain region in which a branch is located, could also be encoded, allowing for explicit alignment by anatomical domain. While we found no association between axonal consensuses and spatial location relative to the soma (e.g. Figure 5c and e), such associations may be anticipated with apical dendrites and more complete axonal reconstructions spanning multiple brain regions, and spatial encoding would facilitate such discoveries. The code available at http://krasnow1.gmu.edu/cn3/NeuriteSequence/ includes sample expanded branch feature encoding to enable future exploration by the research community.

An extended encoding could also improve classification. While we showed that the alignment space is at least as informative as standard topological metrics, whole-neurite metrics (e.g. height, total length, convex hull volume) and branch level features such as length and tortuosity are also useful for classification. For a discussion of which morphometrics are effective for neuronal classification, see [45]. A feature matrix composed of alignment space dimensions and morphometrics would allow for classification on both. In cases in which classes are similar in terms of average branch level metric but different in their relation to topology, the extended encoding has the potential to be more powerful than simply adding branch level metric distributions to the feature matrix. An optimized multi-stage classification which aligns only neurites similar by standard morphometrics could deliver an improved classifier with only a modest increase in alignment computation.

### Optimizing algorithms and analysis

The sequence approach depends critically on the constraint that small-side subtrees necessarily be matched to other smaller-side subtrees. This condition produces near-optimal results (equivalent to the tree edit distance) with moderately to highly asymmetric trees, which have low probabilities of improved tree-alignment from a larger-side subtree aligned to a smaller-side subtree. The scores are near optimal even in the case of trees with greater symmetry, so long as the subtrees are topologically similar. This assumption is generally true of neurites, which explains its effectiveness in this study. However, it is possible to conceive a “worst-case scenario” of a root bifurcating into two large subtrees with exactly the same number of branches, but one fully symmetric and one fully asymmetric. Aligning the corresponding sequence with an almost identical arbor in which a single branch is added to the symmetric side (or a single *C* node removed from the asymmetric side) would yield a dismally low score despite the great structural similarity between the two trees.

To gauge the risk incidence of this effect in neurites, we assessed the asymmetry characteristics of bifurcations with similar subtree sizes, specifically, those with a difference of no more than 6 bifurcations. Of the 5% of axon, 26% of apical dendrite, and 36% of dendrite bifurcations satisfying the condition, the median difference in asymmetry was 0.14, with 75% having a difference less than 0.25 (95% with a difference less than 0.47), though the differences were much smaller for the 5% of subtrees with more than 50 bifurcations. This survey suggests that only dendrites might be susceptible to this problem, though with reasonably small impacts for each instance. Thus, based on currently available data, the relatively simple topological encoding results in practically robust sequence alignments However, the possibility that future larger dataset of complete axonal reconstructions will require a more optimal alignment method cannot be ruled out.

In the big-data prospect of orders-of-magnitude increase in neurite dataset size, heuristics will become important for efficient processing. Given that MDS does not require distances for every neurite pair, not all pairwise combinations are necessary for producing an alignment space. Moreover, for search or analysis, topological metrics can be used to limit the number of neurite alignments by filtering out those that would likely score poorly, as achieved by BlastNeuron with a broader range of morphometrics [15]. The PASTA algorithm itself can be sped up by ignoring the possibilities least likely to contribute to an optimal alignment (e.g. those corresponding to the upper right and lower left corners in the alignment matrix: see Additional file 1: Figure S1c). Other heuristic approaches could be applied that involve candidate seed locations of short matching strings from which the alignment is extended in either direction [46]. The most efficient modern algorithms index seeds in advance [47]. A larger dataset of more complete axons, widely expected from ongoing and discussed connectomic projects, would require appropriate heuristics and efficient implementation in a compiled language, such as C or C++. In cases where sensitivity is more important than speed, sequences of branch feature vectors could take the place of discrete characters.

The diversity of available gene sequence alignment tools highlights the utility of specific parameters and alternative algorithms for different experimental questions or computational requirements [47-50]. Methods of neurite pairwise alignment of one sort or another, including variations on our method, will likely grow in a similar manner to fill various niches, whether in terms of sensitivity or efficiency.

## Conclusion

The newly developed method for aligning binary tree sequences enabled the comparison of large numbers of neurites and revealed their shared topological features. Sequence alignment similarity with a purely topological encoding was sufficient to distinguish different arbor types (axons, dendrites, and apical dendrites) as well as certain neuron types, brain regions, and species. Moreover, the sequence representations could differentiate cell classes better than (or at par with) traditional morphometrics and are consistently as informative as traditional topological metrics. Cluster analysis produced groupings highly associated with known metadata differences, and multiple sequence alignment generated a consensus representation of each cluster that revealed the common topological features. Comparative analysis between consensus and motif profile demonstrated that these analyses captured complementary topological characteristics, with consensuses reliably extracting global features and motifs effectively quantifying local features.

## Additional file

Additional file 1:
**This file provides additional methodological information regarding (1) alignment score baselines and distance normalization, (2) non-metric multidimensional scaling, (3) model-based clustering, (4) cluster-metadata associations, and (5) classification.** It additionally includes four supplementary figures referenced in the main text.
