# Supplementary material for: Topological characterization of neuronal arbor morphology via sequence representation: II - global alignment
Source: BMC Bioinformatics. 2015 Jul 4;16(1):209. doi: 10.1186/s12859-015-0605-1 (PMC4491275; doi:10.1186/s12859-015-0605-1)
Supplement: Additional file 1: — This file provides additional methodological information regarding (1) alignment score baselines and distance normalization, (2) non-metric multidimensional scaling, (3) model-based clustering, (4) cluster-metadata associations, and (5) classification. It additionally includes four supplementary figures referenced in the main text. [file 12859_2015_605_MOESM1_ESM.pdf]

Supplementary Material

SM.1 Score baseline and distance normalization

Each alignment score was normalized to a baseline of alignment scores given random trees of the same sizes of the neurite sequence pair. For each sampled length, 1,000 tree shapes were generated randomly and encoded as sequences. Sequence alignment was performed between ordered sequences for each length pair such that the *n*th sequence of length L_1 was aligned with the *n*th sequence of length L_2 to avoid any dependence between scores, resulting in 1,000 scores for each length pair. Each score was then converted to a per-character score (S_{pc}) based on the length of the shorter sequence. This was achieved by adding back to the score the length difference of the two sequences, as well as the value of one gap opening (G), and dividing by the shorter sequence length. An optimal per-character score would be 1 and the lower bound for the score is limited only by the size of the sequences and the potential number of gaps and gap openings. With no gap open cost the minimum per character score would be -1.

$$S_{pc} = \frac{S + |L_1 - L_2| + G}{min(L_1, L_2)}$$

The mean and standard deviation of those per-character scores was taken for each length pair. Means and standard deviations of length pairs falling between those sampled were interpolated. All real neurite per-character scores were converted to z-scores based on the per-character score mean and standard deviation of the neurite pair's lengths (**Supplementary Figure 2**). Each z-score was converted back into a per-character score using a set mean and standard deviation, creating a fully normalized score (S_{norm}). The mean and standard deviation were chosen such that the probability of a perfect per-character score of 1 would be exceptionally low. Finally, the fully normalized scores were converted to distances by subtracting each score from the optimal score of 1. In the rare case of a fully normalized score above 1, resulting in a distance value less than 0, an exponential function was applied making the distance greater than 0. Baseline score normalization was calculated in R prior to further analysis.

SM.2 Non-metric MDS

Non-metric MDS is performed by embedding (usually by the standard MDS algorithm) then iteratively shifting element location until *stress* is minimized. *Stress* is the measure of how different each neurite pair's position is between the original ordered list of distances and the ordered list derived from the embedded space. Thus, the two neurites with the smallest distance between them in the original data should also be the two nearest neurites in the new space, while two neurites that were a median distance apart in the original data will be about a median distance apart in the new space relative to other neurite pairs.

The *MASS* [23] R package was used to perform non-metric MDS on datasets consisting of all neurites and separate instances of the data composed of neurites from each arbor type. In each case a sufficient number of dimensions were used such that the stress was at or below 15% and was decreasing by less than 1% with each additional dimension. Inspection of the spaces showed no effective differences in the

primary dimensions used for analysis between spaces generated using more dimensions. Moreover, the spaces produced for each arbor type were very consistent with the space produced for all arbors.

SM.3 Model-based clustering

We used the *mclust* [26, 27] R package for model-based clustering, which performs Expectation Maximization (EM; [28]) for generating optimal parameter values in order to fit the data with a set of multivariate Gaussian models. Variable Gaussian features include size, shape (different size in each dimension), and orientation. Shape and orientation may be unspecified, resulting in spherical clusters (similar to k-means clustering), or ellipsoidal clusters without orientation. Each parameter type could be equivalent or variable across clusters. These options are not exhaustive, but for the purposes of exploration they are effective and provide a useful limit on an otherwise unlimited set of models. Multiple parameter sets and number of clusters were tried and a Bayesian Information Criterion (BIC) value produced for each. The BIC quantifies the optimal balance between the predictive power of a model (measured by the log of its likelihood given the data) and its complexity (in terms of number of parameters *k* and the log of dataset size *n*):

$$BIC = \ln(L) - k * \ln(n)$$

SM.4 Cluster-metadata associations

To quantify the association between clusters and metadata groups with by χ^2 test, the expected counts were computed from the marginals of the contingency table. The standardized residuals (indicating how much any given cluster-group pair contributed to the result) were converted into raw p-values; these were then adjusted with Bonferroni-correction using the degrees of freedom (N_C - 1) x (N_G - 1), where N_C and N_G are the number of clusters and metadata groups, respectively. Cases in which the count in a cell was higher than the expected value and in which the corrected p-value was less than 0.05 indicated a statistical overrepresentation, and thus an association between the cell's cluster and group.

SM.5 Classification

In order to maximize classification performance and determine the most informative features, clustering was run on all possible permutations of alignment space dimensions and all possible permutations of topological metric principal components for a specified pair of neurite groups. For two-class discrimination (e.g. CA3 and CA1 pyramidal neurons) models were fixed to two clusters and alignment space Gaussians were limited to equal size and no orientation.

LDA and feature selection were respectively performed by the R packages *MASS* [23] and *klaR* [29]. Variables could be added or removed in the selection process and the threshold for updating the model was a 1% improvement in accuracy. A 10-fold cross-validation was used, leaving 10% of the data for testing the accuracy of the final model. Given the stochastic nature of data division, different models were generated with non-identical accuracy values in multiple runs. The process was run 10 times for each case and the accuracy averaged. The optimal models were found most times and always provided the final accuracy value. The order of mean, median, and max accuracy scores between variable sets (alignment space and topological metric principal components) were consistent.

Supplementary Figures

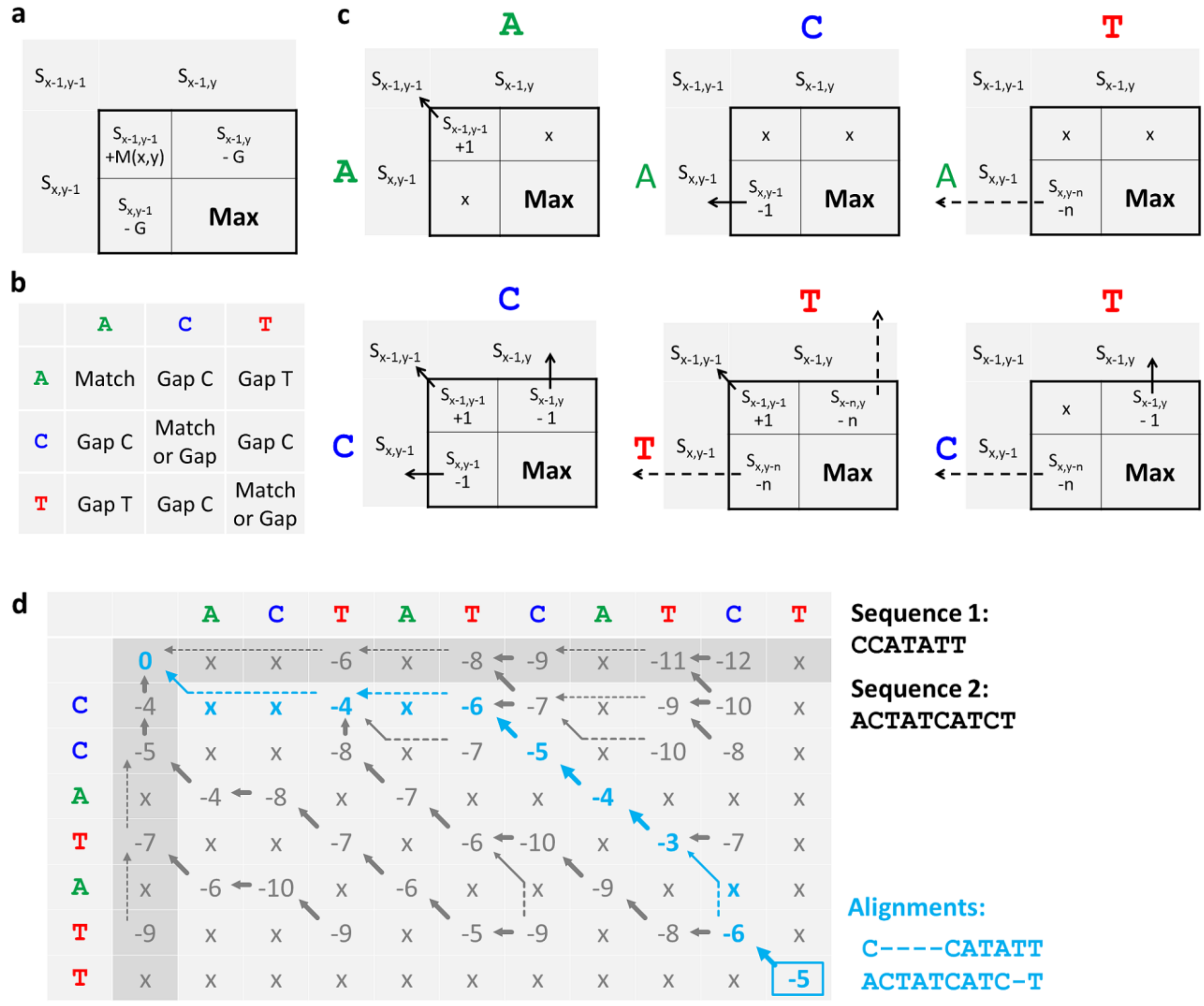

Supplementary Figure 1: Dynamic programming alignment of tree node types. a. The first table shows the generic calculation of scores for a given matrix position (x,y) using the scores of cells to the left, top, and top-left. The score from the top-left is added to the match score for the characters at (x,y). The score for gapping from above equals the score above (x-1,y) minus the gap penalty, while the score for gapping from the left is that score (x,y-1) minus the gap penalty. The cell's score is then the maximum value of the match and gap scores. **b.** Cells with certain node type pairs have restrictions as to whether a match, left-gap, or right-gap is allowed. **c.** For each node type pair, arrows point to where the scores are originating from. Dashed arrows for gapping a T node represent a multiple position gap based on the requirement that the gap continue until the start of the node's subtree. **d.** In this complete alignment matrix for the shown sequences, the blue path represents the optimal back-trace. The backward arrows of every cell with a score lead back to the preceding cell that produced the optimal score. Arrows with dashes followed by a diagonal solid line represent an alignment of a C node with a larger subtree, such that the gap begins at a T and back-traces to an A that matches the C.

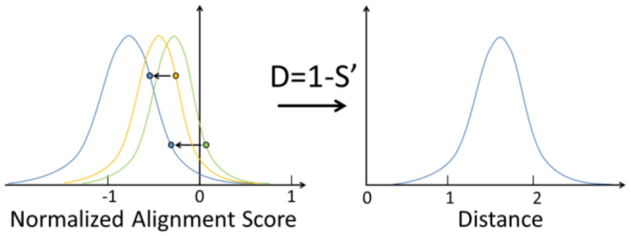

Supplementary Figure 2: Normalizing the per-character alignment score. A given per-character alignment score has a z-score with respect to the distribution of baseline scores for the specific length pair of the aligned sequences. The yellow and green points represent scores from alignments with different length pairs. The lines represent the baseline distribution of per-character scores. Each point is then normalized (arrows) using their respective distribution to the mapped value along an arbitrary length pair distribution (blue points). The distance values are calculated by subtracting the normalized per-character scores from the maximum possible score of 1.

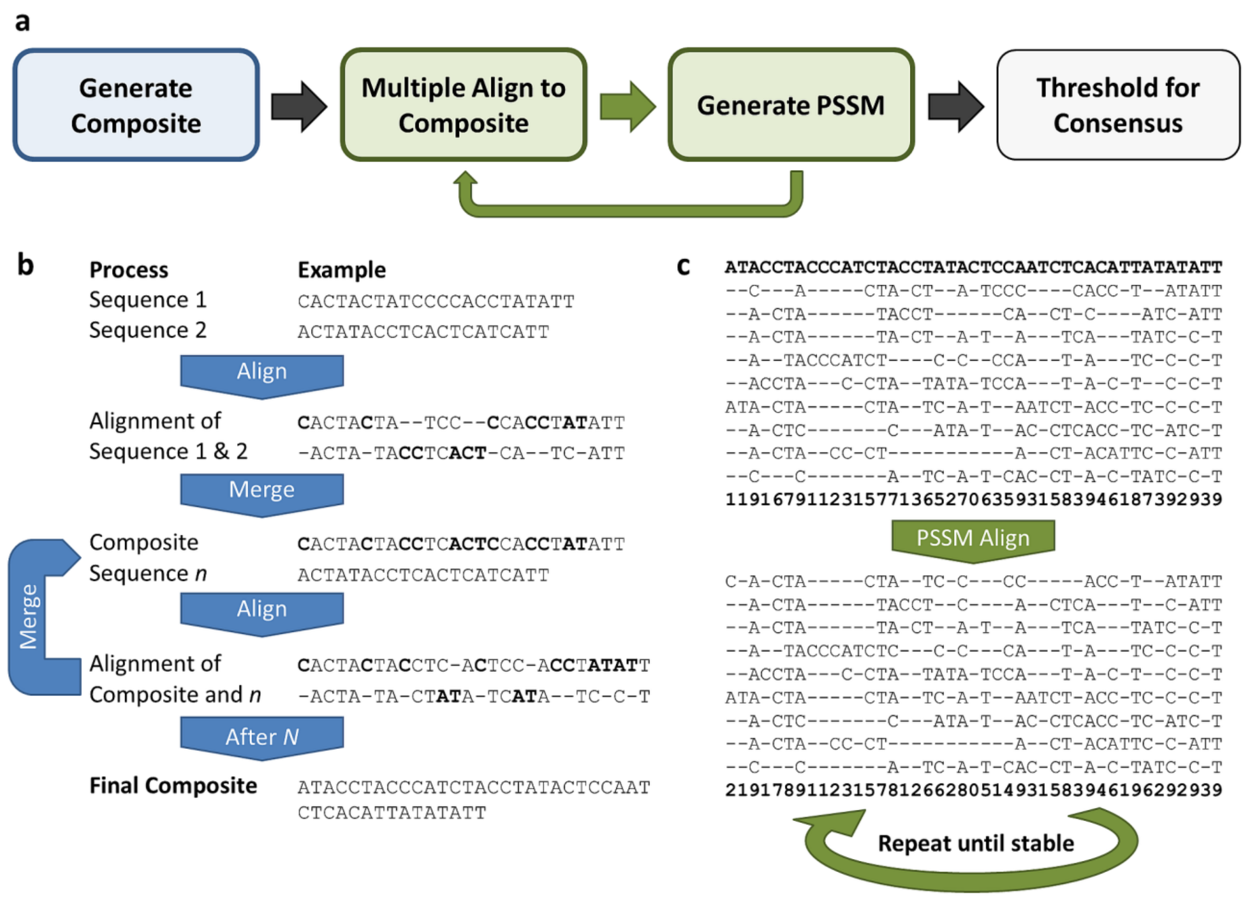

Supplementary Figure 3: Multiple sequence alignment algorithm. a. Flow chart of the process, ending in a multiple alignment and a threshold-defined consensus. **b.** Construction of a composite begins with the matching of two sequences from the set to be aligned. The alignment of those sequences is merged into the composite which is then aligned to the next sequence. The process repeats until all *N* sequences have been merged into the composite, forming the final composite. **c.** All sequences are aligned to the final composite, creating a multiple alignment. A position-specific scoring matrix (PSSM) is generated based on the conservation of each position (i.e. how many sequences align at the position). The sequences are again aligned to the composite, but this time the score of a match is determined by the PSSM. The process repeats until the multiple sequence alignment is stable.

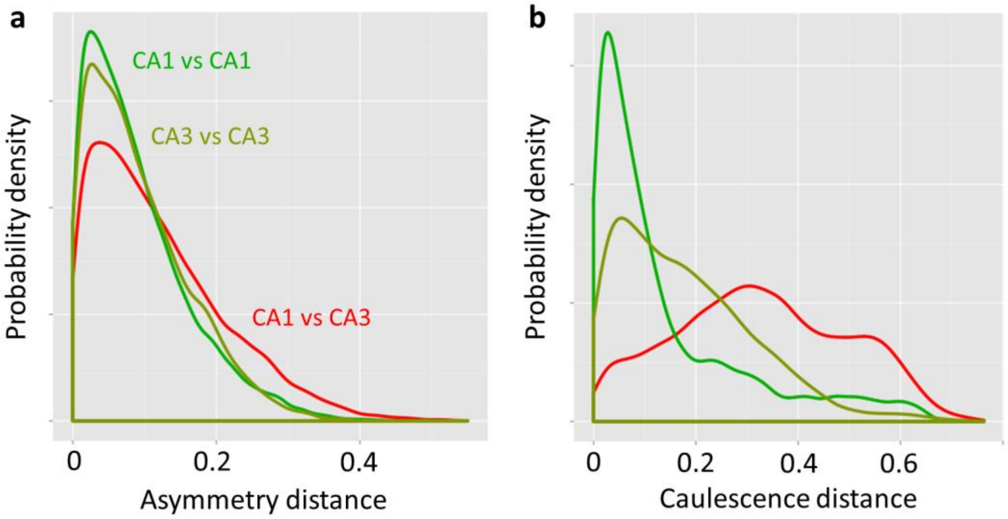

Supplementary Figure 4: Within and between-class distances. Distributions of distances within and between hippocampal CA1 and CA3 apical dendrite classes based on (**a**) average partition asymmetry and (**b**) caulescence.
